# Imaging in Percutaneous Coronary Intervention

**DOI:** 10.31083/j.rcm2306204

**Published:** 2022-05-31

**Authors:** Mohan Satish, Anastasios Roumeliotis, David Power, Anton Camaj, Johny Nicolas, Daniel Feldman, Davis Jones, Keisuke Yasumura, Frans Beerkens, Saman Suleman, George Dangas

**Affiliations:** ^1^Zena and Michael A. Wiener Cardiovascular Institute, Icahn School of Medicine at Mount Sinai, New York, NY 10029, USA; ^2^Department of Medicine, Icahn School of Medicine at Mount Sinai, New York, NY 10029, USA; ^3^Department of Medicine, Donald and Barbara Zucker School of Medicine at Hofstra/Northwell, Hempstead, NY 11549, USA

**Keywords:** intracoronary imaging, optical coherence tomography, intravascular ultrasound

## Abstract

Intracoronary imaging (ICI) use during percutaneous coronary intervention (PCI) has been shown to effectively improve cardiovascular outcomes, particularly for high-risk subgroups. However, data from randomized controlled trials are limited and the overall utilization rate of ICI remains variable between different countries and centers. Potential benefits of ICI include identification of appropriate lesions for PCI, improved characterization of lesions, and optimization of stent placement. Currently available modalities of ICI include intravascular ultrasound, optical coherence tomography and near infrared spectroscopy. Within this review, we summarize the contemporary evidence surrounding ICI and discuss its application in clinical practice.

## I. Introduction 

Coronary angiography allows for the assessment of luminal diameter and a relative assessment of coronary stenosis. However, the angiographic evaluation of coronary artery disease has numerous limitations. Most importantly, the two-dimensional visualization of the coronary vessel may confine accurate assessment of lesion characteristics and lead to suboptimal subsequent stent placement [1, 2]. Intracoronary imaging (ICI) techniques like intravascular ultrasound (IVUS) or optical coherence tomography (OCT) can provide helpful information on lesion characteristics during procedure planning and assist with optimal stent placement [3]. Nevertheless, utilization of ICI remains low with high variability across different centers [4]. This may be driven by limitations in cost, expertise, and availability [5]. Herein, we review the current evidence on ICI as it applies to procedural aspects and specific clinical subgroups, and critically discuss future directions.

## 2. Mechanics of Intracoronary Imaging 

The main modalities of ICI (IVUS and OCT) differ significantly in their mechanisms (Table 1). While IVUS utilizes ultrasound waves formed by the oscillatory movement of a transducer as the source of image production [6], OCT utilizes near-infrared light for intracoronary visualization, creating a bloodless field by high velocity contrast injection for rapid lumen imaging acquisition. Contemporary iterations of OCT now utilize frequency domain (FD) imaging which utilizes high viscosity liquids to the same end [7, 8]. Coronary angiography is generally required for both imaging modalities. For coronary artery access with IVUS, a transducer is attached to a guide catheter (a minimum of 5 Fr) and luminal measurements are obtained by manual or motorized pullback upon vessel entry [8]. The axial and lateral resolution of greyscale IVUS is 100–150 μm and 200 μm respectively with a penetration depth of 4–8 mm [6]. To this effect, a significant IVUS-derived parameter is the minimal lumen area (MLA) as it facilitates functional evaluation of a lesion. In contrast, specialized OCT catheters consisting of optical fibers with a hollow metal wire torque, are utilized with higher acquisition speeds (25 mm/s) than that of IVUS upon coronary vessel entry [8]. The axial and lateral resolution of OCT are both much greater than that of IVUS, at 10–20 μm, and 20 μm respectively; however, this comes at the expense of a lower penetration depth of 2 mm [9].

**Table 1.** **Mechanics of Intracoronary Imaging**.

Modality:	**OCT**	**IVUS**
Mechanism:	near-infrared light	ultrasound waves
Penetration:	2 mm	4–8 mm
Resolution:	10–20 mm (axial)	100–150 mm (axial)
	20 mm (lateral)	200 mm (lateral)
Advantages:	plaque characteristics	vessel wall remodeling
	stent changes, post-PCI	calcifications
Limitations:	requires a bloodless field	discernment of layers of coronary vessel wall
Image acquisition (normal coronary vessel anatomy shown):	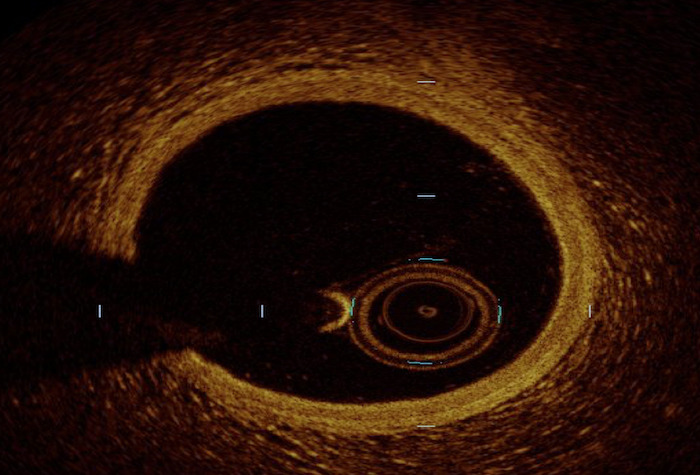	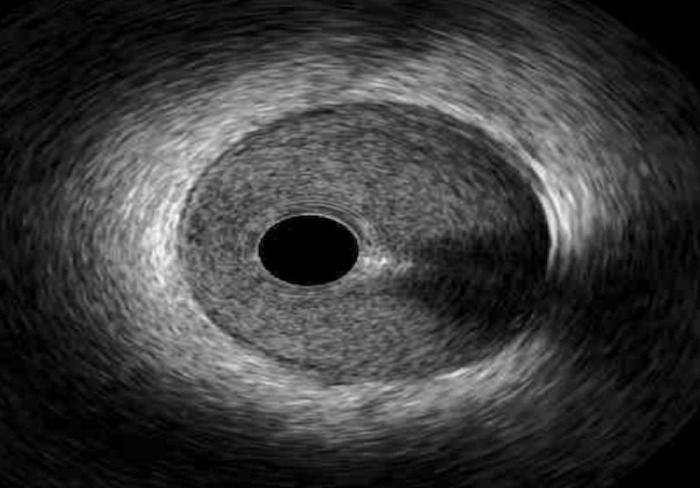

The differences in penetration and resolution explain the inherent limitations of both modalities in comparison. Given its relative limited resolution, IVUS is unable to evaluate the separation of the vessel wall layers (i.e., intima, media and adventitia) compared to OCT [10]. Therefore, OCT is better served within reasonable penetration (<1–1.5 mm) for evaluation of plaque characteristics, including vulnerable plaque markers (i.e., thin-cap fibroatheroma and neovascularization), intrinsic vessel wall characteristics and post-PCI stent changes [11, 12, 13, 14]. Conversely, OCT is limited by its penetration compared with IVUS; therefore, IVUS is more suitable for assessing vessel wall remodeling patterns and identifying dense materials such as calcium that reflect more ultrasound waves [15, 16, 17, 18].

Lastly, irrespective of considerations on penetration and resolution, the need for a bloodless field by high velocity contrast injection with OCT carries two pertinent limitations. First, the identification of aorto-ostial lesions is limited by such a need with OCT, and second, the use of OCT requires the use of contrast and inherently carries a risk of contrast induced nephropathy, particularly for patients with renal impairment [8]. Considering these parameters, the safety and feasibility of both modalities have been shown to be comparable in observational studies [19, 20].

## 3. Utility of Intracoronary Imaging 

**Fig. 1.**
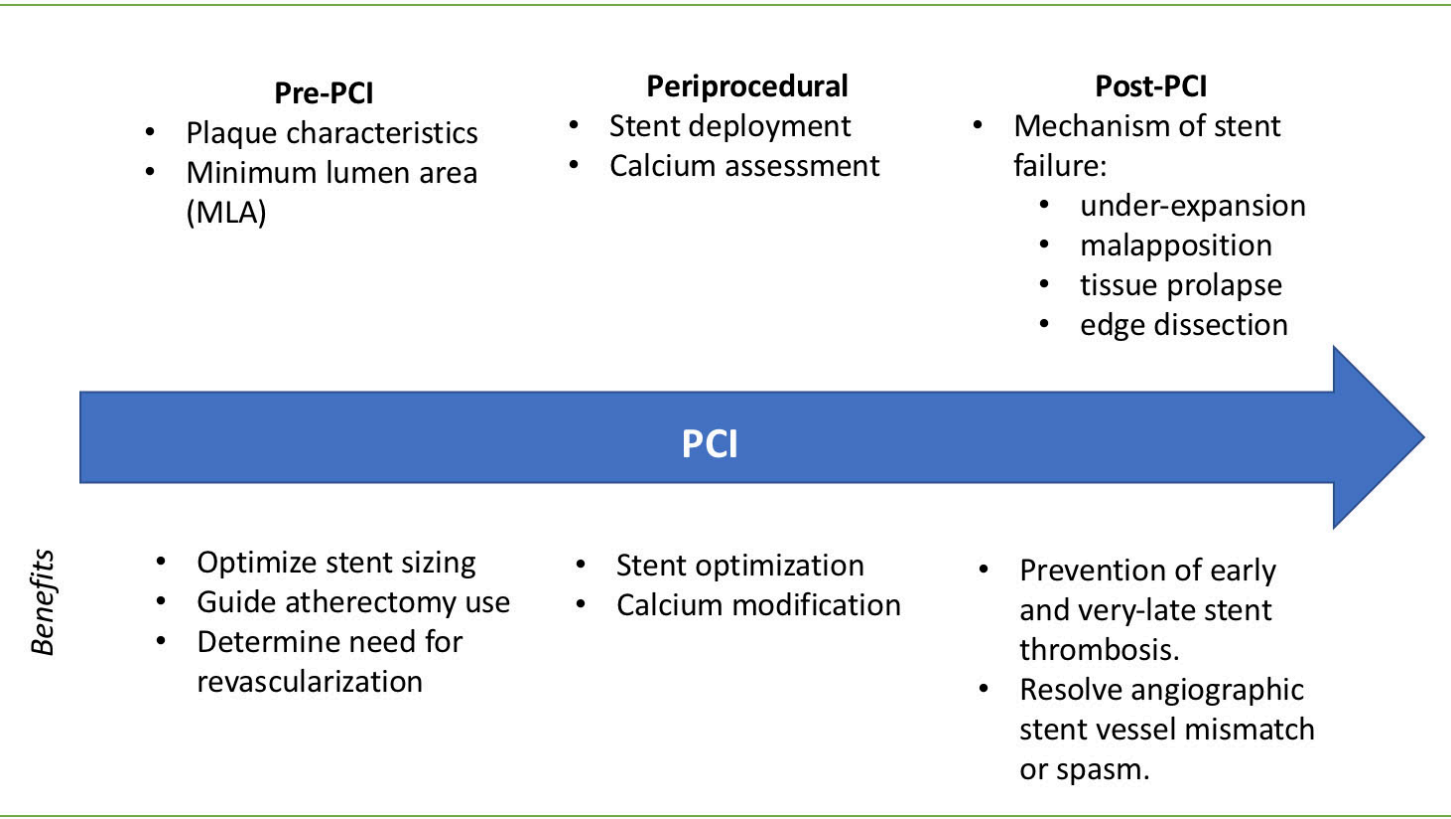
**Utility of Intracoronary Imaging in Percutaneous Coronary Imaging (PCI)**. Components of intracoronary imaging assessment (above) to guide decision-making (below) at each stage of PCI.

**Fig. 2.**
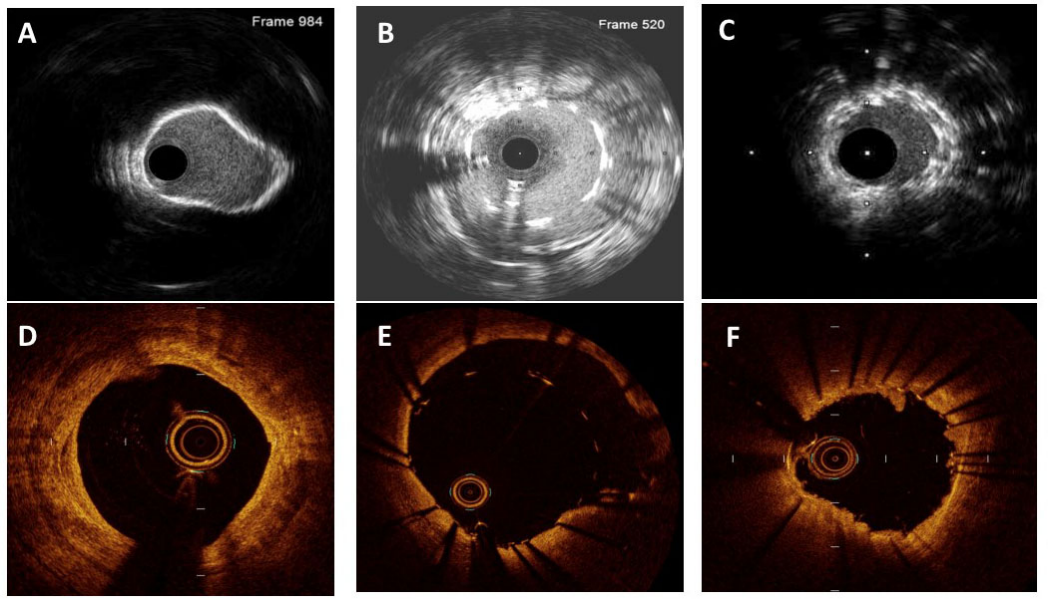
**Lesion and Stent Failure Identified by Intracoronary Imaging**. (A & D) Calcified plaque by IVUS and OCT, respectively. (B & E) Stent malapposition by IVUS and OCT, respectively. (C) Stent under-expansion by IVUS. (F) Tissue prolapse by OCT.

The use of ICI during PCI can inform pre-, peri- and postprocedural decision-making (Fig. 1). The pre- and periprocedural use of IVUS or OCT can help with planning for appropriate lesion preparation and modification, in addition to deciding on optimal stent size. Following stent implantation, ICI can assist the operator to achieve optimal stent apposition in addition to further assessing the risk of stent failure (Fig. 2).

### 3.1 Pre- and Peri-PCI 

Pre- and periprocedural assessment using ICI includes evaluating for calcium and characterizing plaque. With these considerations, assessment of significant stenosis and determining optimal stent sizing are also accomplished. The detection of calcium by angiography appears comparable to IVUS and OCT, while calcified plaque is better assessed with ICI [1, 21, 22, 23, 24]. This is important since a higher risk for stent under-expansion has been observed with calcium angles >180° by both OCT and IVUS [25, 26]. Therefore, one of the main reasons for the use of ICI is to evaluate the need for calcium modification by rotational or orbital atherectomy, or for the use of scoring or cutting balloons [24, 25]. Subsequently, stent expansion on deployment is mediated by the effective calcium modification. Furthermore, attenuated (hypoechoic plaque with deep ultrasound attenuation) or lipid-rich plaques identified by ICI have been associated with worse outcomes during PCI [26, 27, 28, 29, 30, 31]. For example, identification of plaque lesions with a large necrotic core by ICI may benefit from use of distal embolic protection devices during PCI [30]. Furthermore, stent under-expansion predicts early stent thrombosis and restenosis, highlighting the need for optimal stent sizing [1, 32, 33, 34, 35]. Compared to angiography, OCT or IVUS guidance provides larger measurements by a luminal approach (e.g., MLA) and therefore allows for the use of stents with larger diameters [36, 37, 38, 39]. Therefore, currently the use of MLA by IVUS remains as one of the most important ICI parameters to objectively identify significant lesions to guide a decision for revascularization. Compared to the gold standard approach for ischemic assessment by fractional flow reserve (FFR), ICI derived measurements have correlated poorly with FFR among angiographic intermediate stenosis but may augment functional assessment in difficult anatomic lesions (aorto-ostial, tandem, LM) [40].

### 3.2 Post-PCI 

Postprocedural assessment with ICI has the potential to identify immediate post-PCI complications that can lead to stent failure by either restenosis or stent thrombosis, including stent under-expansion, malapposition, tissue prolapse, and edge dissection. By identifying the mechanism of stent failure, ICI can help guide further management.

ICI carries a class IIa recommendation by the European guidelines for the evaluation of stent failure [41]. Appropriate determination of stent expansion by ICI measurements of the reference lumen area and minimum stent area (MSA via IVUS) may prevent adverse PCI events from stent under-expansion, particularly early and very late stent thrombosis [24, 42, 43, 44, 45]. Stemming from this, the European Society of Cardiology (ESC) supports the clinical adoption of achieving a stent expansion by an MSA that is at least 80% of the reference lumen area [1].

Among mechanisms of stent failure, malapposition refers to inadequate stent strut contact with the vessel wall and is better identified by OCT compared with IVUS [46, 47, 48]. Evidence for adverse outcomes due to stent malapposition remains mixed [49, 50]. Notably in comparison, significant stent under-expansion has repeatedly been identified as one of the strongest risk factors for early stent thrombosis and therefore stent failure [51]. Interestingly, a unique parameter of OCT evaluation of adequate stent expansion requires proximal and distal stent dilatation that is at least 90% of a given reference segment [41]. By identifying stent under-expansion, application of high pressure balloons can be utilized during index or repeat revascularization. Similarly, tissue prolapse, where there is extrusion of plaque from outside the stent area, and stent edge dissections of the vessel wall, are both findings better diagnosed with OCT that increase the risk of early stent thrombosis but can resolve angiographic misdiagnoses of stent vessel mismatch or spasms [41, 52, 53, 54, 55]. Furthermore the utility of ICI with stent failure due to restenosis extends to the capacity of OCT to evaluate for contributory stent fracture or neoatherosclerosis [56, 57]. The greater spatial resolution of OCT facilitates a higher identification of neointimal rupture and thrombi which appear contributory to the identification of neoatherosclerotic plaque where repeat stenting may be necessary.

## 4. Intracoronary Imaging versus Angiography 

The use of IVUS for PCI guidance has demonstrated a benefit compared to coronary angiography alone in the bare metal stent (BMS) era. This finding was largely due to reductions in target vessel revascularization (TVR) and restenosis, but with neutral findings on mortality and myocardial infarction (MI) [18, 58, 59, 60]. In the drug-eluting stent (DES) era, observational and randomized controlled trial (RCT) data have confirmed these findings, particularly for patients with complex lesions including long lesions (implanted stent >28–30 mm), bifurcation disease, chronic total occlusions (CTOs), and unprotected LM disease [61, 62, 63, 64, 65, 66, 67, 68, 69, 70, 71, 72, 73, 74, 75].

### 4.1 Societal Guidelines

The earliest success with ICI was first realized in an attempt to defer systemic anticoagulation with stent placement by achieving adequate stent expansion via IVUS [76]. Currently, European Society of Cardiology (ESC)/European Association for Cardio-Thoracic Surgery (EACTS) and American College of Cardiology/American Heart Association/Society for Cardiovascular Angiography & Interventions (ACC/AHA/SCAI) guidelines have class II recommendations for IVUS with varying degrees of evidence, best supported for optimizing stent implantation in select cases, preventing stent failure or restenosis, and angiographically assessing LM lesions [77, 78]. In parallel, current OCT guidelines are similar between ESC/EACTS and ACC/AHA/SCAI recommendations (Class IIa, level B) with support for its use as an alternative for imaging guidance [77, 78].

### 4.2 Randomized Control Trials of ICI

Among RCTs in the DES era that have evaluated the comparison of angiography with IVUS (Table 2, Ref. [70, 72]), the IVUS-XPL (The Impact of Intravascular Ultrasound Guidance on Outcomes of Xience Prime Stents in Long Lesions), CTO-IVUS (Chronic Total Occlusion Interventional with Drug Eluting Stents) and ULTIMATE (Intravascular Ultrasound Guided Drug Eluting Stents Implantation in “All-Comers” Coronary Lesions) trials demonstrated significant reductions in major adverse cardiac events (MACE) or target vessel failure (TVF) [67, 68, 72]. Likewise, in a meta-analysis of RCTs (4724 DES-treated patients), IVUS guidance compared to angiography alone was shown to be associated with significant reductions in MACE, cardiac mortality, TVR, target lesion revascularization (TLR) and definite or probable stent thrombosis compared with angiography alone [75]. However, there were no differences in all-cause mortality or MI between the two groups. Overall, the variability in study design and follow up duration confer limited generalizability of observed findings [67].

**Table 2.** **Summary of Randomized Evidence with Intracoronary Imaging Versus Angiography in the Drug-Eluting Stent Era**.

RCT study:	Year of publication	MACE reduction vs. angiography (Yes/No; * statistical significance)	Pertinent primary cohort characteristics
ULTIMATE	2018	Yes *	Left main disease, long lesions, chronic total occlusions
Zhang *et al*. [72]	2016	Yes	Small vessel diameters
IVUS-XPL	2015	Yes *	Long lesions
CTO-IVUS	2015	Yes *	Chronic total occlusions
AIR-CTO	2015	Yes	Chronic total occlusions
Tan *et al*. [70]	2015	Yes	Unprotected left main disease
RESET	2013	Yes *	Bifurcations, prior PCI
AVIO	2013	Yes *	Bifurcations, long lesions, chronic total occlusions, small vessel diameters
HOME DES	2010	Yes	Insulin-dependent diabetes, left main lesion, in-stent restenosis, long lesions, bifurcations

Prior to the ULTIMATE trial, the lack of an RCT evaluating ICI in an all-comer population conferred limitations to early randomized and registry data. Additionally, a majority of the prior RCTs were limited in power and had lower complexity of CAD [67, 68]. Furthermore, predefined optimization criteria was not ubiquitously applied across these RCTs to increase the probability of utilizing larger MSAs. In an attempt to address both concerns, the ULTIMATE trial was the largest RCT to consider IVUS guidance among 1448 all-comer patients requiring DES implantation [72]. Many differences in comparison to the IVUS-XPL trial existed that include study endpoint definition, loss to follow-up and number of IVUS criteria necessary to be met for optimization. The primary endpoint of TVF at 12 months was lower with IVUS guidance compared to angiography alone among all comers irrespective of lesion complexity in the ULTIMATE trial (hazard ratio 0.53; 95% confidence interval: 0.31–0.90, *p* = 0.019) [72]. Additionally, achievement of IVUS-defined criteria for the optimal stent deployment in the ULTIMATE was associated with further net benefit after subgroup analysis: (1) MLA in stented segment >5.0 ${\mathrm{mm}}^{2}$ or 90% of the MLA at the distal reference segments; (2) plaque burden at the 5 mm proximal or distal to the stent edge less than 50%; (3) no edge dissection involving media with length longer than 3 mm.

Importantly, the feasibility of IVUS-defined criteria can be questioned when only a small percentage of patients achieved the optimal result despite rigorous post-dilatation and effort. However, in the 3-year follow-up data of the ULTIMATE trial, IVUS guidance with PCI showed a persistent significant reduction in TVF compared to angiography, which was again particularly upheld among those meeting IVUS-defined optimization criteria compared to those who did not [79]. Nevertheless, a significant criticism of the ULTIMATE trial is that the complexity of disease was not inclusive enough of a true all-comer population with the utilization of larger stents per patient (average of 66 mm) and a significantly large percent of patients with very complex disease (LM, CTO, etc.).

Evidence supporting IVUS use extends beyond CTOs and long lesions into two specific complex situations - specifically LM disease and in patients with chronic kidney disease (CKD). In a small RCT of 123 elderly patients, the use of IVUS guidance during LM PCI showed a reduction in MACE at 2 years (driven by a reduction in TLR) [70].

Separately, data from both extensive observational and meta-analyses has supported the use of IVUS guidance during LM PCI that has largely been driven by MACE reduction secondary to lower all-cause mortality [61, 80, 81, 82]. Some plausible mechanisms that have been proposed to support these findings include the use of larger stents with better stent expansion, avoidance of two stent techniques, and more stent post-dilatation with IVUS guidance [1]. However, with no parallel reductions in MI or TLR, a possible mechanistic explanation cannot be surmised without further evaluation devoid of possible confounding. In patients with CKD, as previously discussed, IVUS has an advantage over OCT due to lower utilization of contrast. In a small RCT of 83 patients who were deemed high risk for contrast-induced AKI, IVUS guidance was associated with a reduction in intra-procedural contrast volume [83]. These initial findings have led to direction evaluation of IVUS guidance for zero or ultra-low contrast utilization with PCI in patients with advanced CKD with high procedural success observed [84]. Recently, further observational evidence has extended the possibility of zero-contrast PCI via IVUS guidance with complex lesions in renally impaired patients [85].

## 5. Future Directions 

Despite the available body of evidence and the clinical benefit identified in individual complex patient cohorts with ICI, these imaging modalities remain underutilized. Recent data attributes infrequent use to operator concern for perceived time as well as cost constraints [86]. However, the use of IVUS has been shown to be cost-effective with PCI, driven by the prevention of repeat procedures [87]. Additionally, the EVOLVE Short DAPT (dual antiplatelet study) which demonstrated superior DES outcomes with an abbreviated (3-month vs. 12-month) DAPT course, with very low overall rates of ischemic complications, had a high utilization rate of IVUS guidance (nearly 98%) [88]. Therefore, there is a need to both identify the optimal criteria for IVUS guidance and address potential barriers (cost, availability, expertise and procedure length) in order to promote its use. Similarly, the benefits of IVUS guidance in complex contexts (CTO, LM disease, and long lesions) as described here are currently subject to evaluation in the DKCRUSH VIII (Comparison of IVUS-guided With Angiography-guided Double Kissing Crush Stenting Technique for Patients with Complex Coronary Bifurcation Lesions, NCT# 03770650) study.

**Fig. 3.**
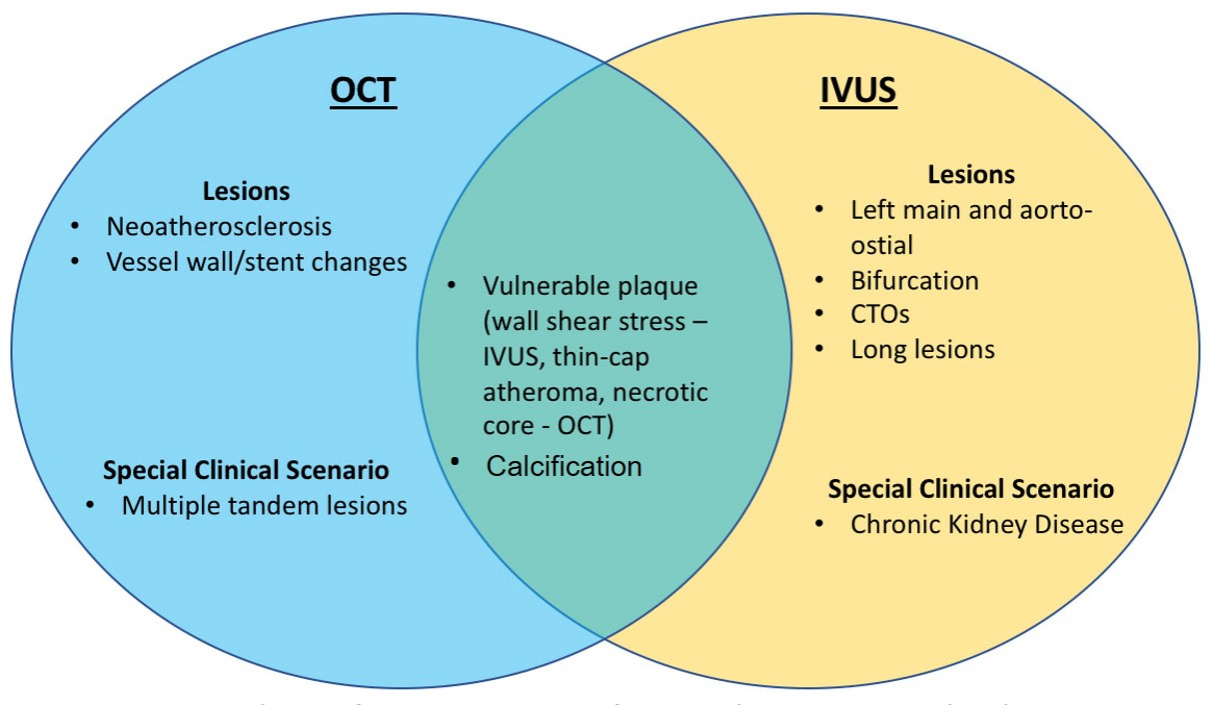
**Selection of Intracoronary Imaging for Lesion Characterization or Clinical Scenario**. Lesion characterization (i.e., chronic total occlusions [CTOs]) and special clinical scenarios are important in consideration of utilizing optical coherence tomography (OCT) or intravascular ultrasound (IVUS).

Furthermore, growing but limited evidence for other modalities of ICI are also available. Prior to the ongoing ILUMIEN-IV trial (OCT compared with Angiography to Guide Coronary Stent Implantation) trial, no previous RCT compared clinical outcomes between OCT guidance and angiography alone with PCI, rather the mere demonstration of superior procedural success [41, 89, 90, 91, 92, 93, 94, 95]. Interestingly, the previous ILUMIEN series of trials also showed non-inferiority to IVUS in post-procedure MSA [41]. As a result, the ILUMIEN IV and OCTOBER (OCT Optimized Bifurcation Event Reduction) trials are ongoing large scale RCTs attempting to demonstrate the superiority of OCT guidance to angiography alone in clinical outcomes during PCI and among high risk patients and/or complex lesions [96]. Nevertheless, identifying the lesion and clinical situations that would benefit with OCT versus IVUS requires further evidence (Fig. 3) [97]. The integration of both ICI modalities and the concomitant use of artificial intelligence (i.e., calculate degrees of calcium) are current explorations for in its advancement [98]. Another ICI modality of growing interest is near-infrared spectroscopy (NIRS) which utilizes electromagnetic radiation with frequencies lower than the visible spectrum to characterize the chemical composition of materials, including tissue [99, 100]. The utility of NIRS has been proposed from observational evidence in combination with IVUS to identify vulnerable plaque, assessing lesion size, predicting periprocedural myocardial infarction and optimizing stent implantation. While currently NIRS has been validated for the detection of vulnerable plaques by the prospective LRP (Lipid-Rich Plaque) study, its utility among these pursuits in a clinical setting requires further assessment, including possible combinations with OCT [101]. Therefore, either improving upon the single imaging capacity of one modality or attempting to combine such modalities are active attempts at overcoming the limitations described here. For example, as described with evaluating lesion severity, physiologic assessment via ICI may be improved upon with the utilization of computational fluid dynamics to simulate coronary flow and pressure. Additionally, along with precise evaluations of plaque composition, assessment of vascular inflammation with ICI, including vessel wall shear stress are ongoing endeavors to further characterize vulnerable plaque.

## 6. Conclusions

The emergence of ICI during PCI is undergoing both a rapid transition in defining clinical application of IVUS and building upon the growing evidence of OCT with complex lesion characterization to guide intervention. It creates new opportunities in the field of interventional cardiology for more accurate lesion assessment and improved post-PCI result. The search of combined ICI approaches to further optimize lesion characterization and stent selection, and the opportunity to improve upon revascularization in vulnerable patients are additional endeavors under investigation. Moving forward, focusing on identifying appropriate populations that would benefit from ICI and lifting the technical and financial barriers will be necessary in order to effectively expand its utilization.
